# Efficacy of a Combination Therapy for Difficulties Waking Up in Non-School-Attending Students

**DOI:** 10.3390/jcm11123271

**Published:** 2022-06-08

**Authors:** Noriyuki Konishi, Hajime Kumagai, Hiroyuki Sawatari, Tetsuro Hoshino, Yoko Murase, Maiko Yamaguchi, Ayako Urabe, Yuka Kiyohara, Aki Arita, Masayo Baku, Ryujiro Sasanabe, Toshiaki Shiomi

**Affiliations:** 1 Department of Sleep Medicine and Sleep Disorders Center, Aichi Medical University Hospital, Nagakute 4801195, Japan; h8k2nk@gmail.com (N.K.); sawatari@hiroshima-u.ac.jp (H.S.); hoshino.tetsurou.299@mail.aichi-med-u.ac.jp (T.H.); yoko_h_1104@yahoo.co.jp (Y.M.); fl_maichan_n@yahoo.co.jp (M.Y.); urabe.ayako.220@mail.aichi-med-u.ac.jp (A.U.); ykiyo@hiroshima-u.ac.jp (Y.K.); akiarita@aichi-med-u.ac.jp (A.A.); mbaku@aichi-med-u.ac.jp (M.B.); sasanabe@aichi-med-u.ac.jp (R.S.); shomi21@hiroshima-u.ac.jp (T.S.); 2 Department of Sleep Medicine, Graduate School of Biomedical and Health Sciences, Hiroshima University, Hiroshima 7348553, Japan; 3 Department of Perioperative and Critical Care Management, Graduate School of Biomedical and Health Sciences, Hiroshima University, Hiroshima 7348553, Japan

**Keywords:** aripiprazole, blue-light exposure, circadian rhythm sleep–wake disorder, long sleep, sleep hygiene guidance, wake-up difficulty

## Abstract

School non-attendance due to difficulties waking up is increasing in Japan, and affected students are commonly diagnosed with orthostatic dysregulation (OD); however, OD-associated sleep problems are overlooked. To date, no sleep-medicine-based treatment for wake-up difficulties in non-school-attending students has been established. This study aimed to assess the efficacy of a novel combination therapy for these students. We assessed the combined effect of sleep hygiene guidance, low-dose aripiprazole administration (3 mg/day), and blue-light exposure on wake-up difficulty in 21 non-school-attending teenage patients. The patients were evaluated using sleep studies and questionnaires before and after treatment. The average subjective total sleep time calculated from sleep diaries before treatment in the patients was 10.3 h. The therapy improved wake-up difficulty by 85.7% and further improved school non-attendance by 66.7%. The subjective sleep time significantly decreased by 9.5 h after treatment (*p* = 0.0004). The self-rating Depression Scale and mental component summary of the 36-item Short-Form Health Survey significantly improved after treatment (*p* = 0.002 and *p* = 0.01, respectively). Wake-up difficulties were caused by the addition of a delayed sleep phase to the patients’ long sleep times. The novel combination therapy was effective in improving wake-up difficulty and mental quality of life in non-school-attending teenage students.

## 1. Introduction

In recent years, difficulties waking up (wake-up difficulties) in teenagers have become a global challenge. A Japanese study reported that the number of students who failed to attend school for >30 days in a year (i.e., “non-school-attending students”) increased to 196,127 in 2020 [1].

Of the non-school-attending students in Japan, 30–40% reportedly had orthostatic dysregulation (OD) [2]. OD is characterized by autonomic nervous system dysfunction, involving various symptoms, such as dizziness and difficulty getting out of bed [2,3,4,5,6,7]. Due to these symptoms, OD is known to cause wake-up difficulty, including the difficulties in waking up and getting out of bed despite being awake. However, in several cases, wake-up difficulty does not improve, even after diagnosis and treatment of OD, possibly due to causes other than OD, such as sleep disorders (e.g., central hypersomnia, circadian rhythm sleep–wake disorder (CRSWD), and sleep insufficiency syndrome). The ineffectiveness of OD treatment for wake-up difficulty indicates that combination treatments are mandatory for these patients.

With respect to treatments for CRSWD, including delayed sleep-phase syndrome (DSPS), previous studies have revealed that sleep hygiene guidance and blue-light (BL) exposure are effective interventions for DSPS [8,9]. In addition to DSPS treatment, low-dose aripiprazole (APZ) administration reduces nocturnal, prolonged sleeping time [10]. Nonetheless, non-school-attending students tend to sleep for considerably longer times than regular students, possibly resulting in wake-up difficulty. Therefore, we attempted to create a new sleep hygiene guidance protocol for long sleepers (10 h sleeper specifications). This study follows our success of combining APZ administration with BL exposure [11].

Hence, this study aimed to assess the efficacy of a newly developed combination therapy for non-school-attending teenage students with wake-up difficulties.

## 2. Materials and Methods

### 2.1. Subjects

A total of 71 non-school-attending patients who were suspected of having hypersomnia visited our sleep center at Aichi Medical University Hospital between February 2014 and March 2018. Among them, 21 teenage patients with complaints of wake-up difficulty due to suspected OD and/or depressive states were included in this single-center, retrospective study. Patients were excluded if they (1) were <13 or >19 years of age, (2) had narcolepsy, and/or (3) had obstructive sleep apnea with an apnea–hypopnea index (AHI) of >10 events/h. This study was approved by the Aichi Medical University Hospital Ethics Committee (approval number 2021–126), and all patients provided informed consent. As this was a retrospective study, an opt-out statement was used for patient consent.

### 2.2. Sleep Diary

All patients were instructed via telephone and fax to maintain a sleep diary at the booking stage for approximately 2–4 weeks before visiting our sleep center. Subjective sleeping time was calculated from each patient’s sleep-diary records. Therefore, the appropriate sleep time for each patient was estimated at the initial visit.

Even after initiating the combination therapy, the sleep diary was maintained to track treatment progress, for example, whether wake-up difficulty and/or school non-attendance improved.

### 2.3. Combination Therapy

**Figure 1:**
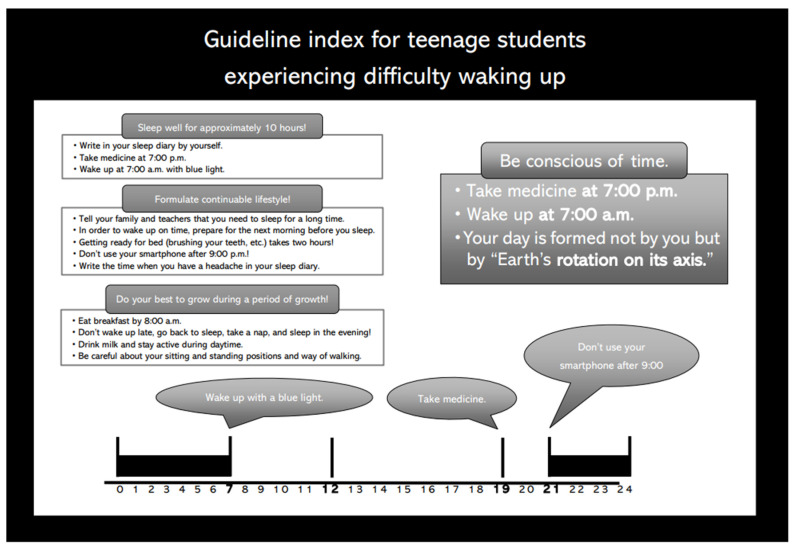
Booklet on sleep hygiene guidance (a sample: 10 h sleeper specifications).

After a thorough explanation of the combination therapy by a medical doctor and consent from the patients and their parents, the 21 teenage patients received sleep hygiene guidance from a certified public psychologist. Appropriate sleep time in daily life was recommended based on the subjective sleep time estimated from each patient’s sleep diary. We informed patients about the importance of having sufficient sleep time in sleep hygiene guidance. Using the booklet we created (sample: 10 h sleeper specification), the sleep hygiene guidance was designed as follows (Figure 1): Each patient took the drug at 7:00 p.m. and stopped using their smartphone at 9:00 p.m. They woke up at 7:00 a.m. and looked at blue light. After making notes in the sleep diary, each patient had breakfast. All patients were also instructed to exercise moderately so as not to take a nap. Based on the estimated subjective sleep time from each patient’s sleep diary, sleep hygiene guidance was provided to each individual, with an appropriate sleep time in daily life recommended using a booklet that we created (a sample: 10 h sleeper specifications) (Figure 1). Additionally, 3 mg/day of APZ was administered at 7:00 p.m. daily to the patients after seeing a doctor. Along with sleep hygiene guidance and APZ administration, patients retired to bed after turning off the lights at 9 pm and received BL exposure (goLITE BLU^TM^, Philips, Netherland) with an alarm clock functioning from 7 am until they woke up.

### 2.4. Sleep Studies

To accurately confirm the diagnosis of hypersomnia, polysomnography (PSG) and the multiple sleep latency test (MSLT) were performed on all patients at our sleep center approximately 1 month after therapeutic intervention. PSG (PSG-1100, NIHON KOHDEN, Tokyo, Japan) included electroencephalography (EEG), electrocardiography, mentalis and lower-limb electromyography, a nasal flow sensor, a chest and abdominal movement sensor for respiratory effort measurement, a blood oxygen saturation monitor, a snoring sensor, and a body position sensor. Additionally, based on the American Academy of Sleep Medicine (AASM) scoring criteria (version 2), a sleep technologist blinded to patient characteristics manually analyzed the recorded data. The recorded data were scored according to five distinct stages: wake, stage N1, stage N2, stage N3, and rapid eye movement sleep. Total sleeping time and sleep efficiency were calculated as sleeping time on EEG and percentage time spent sleeping on EEG after sleep onset, respectively. The MSLT was also performed according to the method recommended by the AASM [12]. Briefly, the MSLT comprises five nap trials, and each trial commenced 2 h after initiating the prior trial. Each trial ended if the patient failed to fall asleep within 20 min. If the patient fell asleep, the nap trial was continued for an additional 15 min. The mean sleep latency on the MSLT was defined as the average time for sleep onset in the nap tests. We also defined hypersomnia as a mean sleep latency ≤8 min in the MSLT.

### 2.5. Assessment Using Self-Administered Questionnaires before and after Treatment

All patients completed the following self-administered questionnaires before and approximately 4 months after the initiation of the combination therapy. The Pittsburgh Sleep Quality Index (PSQI) comprises 19 self-assessment items that assess subjective sleep quality and disability over the preceding month. A higher PSQI score indicates a poorer subjective sleep quality and a greater state of insomnia [13]. The Epworth Sleepiness Scale (ESS) comprises eight self-administered items that measure excessive daytime sleepiness. A higher ESS score indicates stronger excessive daytime sleepiness [14]. The Self-rating Depression Scale (SDS) was used to screen patients for the presence of potential depressive disorders. The SDS scale ranges from 20 to 80 points, with ≥40 points suggesting depressive states [15].

Quality of life (QOL) was evaluated using the Japanese version of the 36-item Short-Form Health Survey (SF-36) [16]. This is a comprehensive measure of QOL, including physical and mental health. It comprises 36 questions that assess eight health concepts. The eight subscales include a physical component summary (PCS) comprising (1) physical functioning (PF), (2) role-physical (RP), (3) bodily pain (BP), and (4) general health (GH), as well as a mental component summary (MCS) comprising (5) vitality (VT), (6) social functioning (SF), (7) role-emotional (RE), and (8) mental health (MH).

### 2.6. Statistical Analysis

Values are presented as the mean ± SD or number (%). Post-treatment changes in subjective sleep quality, depressive symptoms, and QOL were evaluated using a paired t-test or Wilcoxon rank-sum test after the Shapiro–Wilk test. A two-tailed priori *p*-value < 0.05 was considered significant. All statistical analyses were performed using STATA (version.15; Stata-Corp., College Station, TX, USA).

## 3. Results

### 3.1. Pre-Combination Therapy Demographics

The demographics of the 21 teenage patients who underwent PSG, MSLT, and questionnaire surveys are shown in Table 1 and Table 2. The average age, number of male patients, and prevalence of attention-deficit hyperactivity disorder were 14.8 years, 9 (42.9%), and 14.3%, respectively. The most frequently attended school levels were junior high school (71.4%), followed by high school (28.6%).

Regarding the subjective symptoms at the initial visit (Table 1), six patients (28.6%) had excessive daytime sleepiness (EDS) with an ESS score ≥11. Regarding the PSQI, an indicator of insomnia, 12 patients (57.1%) had high values ≥5.5 points. Twenty patients (95.2%) had SDS scores ≥40 points, suggesting depressive states, and the remaining one had an SDS score of 39 points. The average subjective total sleep time calculated from sleep diaries for approximately 1 month before treatment in the 21 patients was 10.3 h. The details of sleep time are presented in Table 1. All 21 patients had delayed sleep–wake phase disorders, and 18 patients had subjective sleeping times of 10 h or more.

The diagnostic results of 21 patients after sleep studies are shown in Table 2. Regarding PSG-related data, sleep structures were within the normal range, including total sleep time and sleep efficiency (Table 2). Regarding the AHI, two patients (9.5%) had 5 ≤ AHI < 10/h (i.e., mild-sleep-disordered breathing); however, no patient had an AHI ≥ 10. No patients had a periodic limb movement index ≥ 5/h. In terms of MSLT-related data, the average sleep latency value was 10.1 min, and sleep-onset rapid eye movement periods (SOREMPs) were observed only once in two patients (9.5%). Six patients (28.6%) who had EDS with an ESS score ≥ 11 met the diagnostic criteria for idiopathic hypersomnia, with a mean sleep latency ≤ 8 min.

### 3.2. Post-Combination Therapy Changes

**Figure 2:**
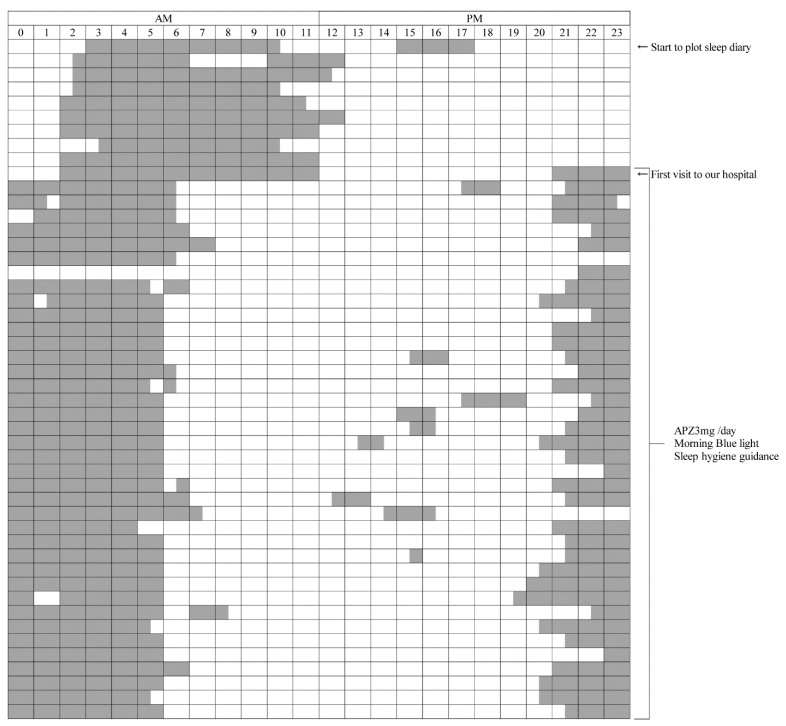
Sleep diary.

After the 21 teenage patients underwent treatment, the incidences of wake-up difficulty and school non-attendance decreased to three (14.3%) and seven (33.3%) patients, respectively (Table 3). In other words, improved wake-up difficulty and school non-attendance were confirmed in 18 (85.7%) and 14 (66.7%) patients, respectively. The subjective average bedtime and wake-up times were 0:06 am and 10:30 am before treatment, but they were earlier after treatment, at 22:00 p.m. and 7:42 am, respectively (both *p* < 0.0001). A 13-year-old male patient’s sleep diary is shown in Figure 2. The subjective average total sleeping time according to the sleep-diary records significantly decreased after treatment (10.3 ± 1.0 vs. 9.5 ± 0.6 h, *p* = 0.0004), while the PSQI and ESS did not improve after treatment (*p* = 0.07 and *p* = 0.77, respectively) (Table 3). Notwithstanding, SDS values decreased significantly after treatment (48.0 ± 6.6 vs. 43.5 ± 7.2 points, *p* = 0.002), indicating improved subjective depressive states. Adverse events of APZ are generally reported to have a 0.1% frequency of malignant syndrome, tardive dyskinesia, paralytic ileus, rhabdomyolysis, and leukopenia. However, in this study, these adverse events were not observed; no serious side effects were observed, and only two patients had mild nausea for several days after the start of oral administration.

Both the left and right ends indicate 0:00 a.m. The gray bars indicate the daily sleep time.

(A) The upper part is the period before the initial visit to our hospital. In the period before treatment, the sleep diary shows a sleep rhythm of DSPS, such as going to bed around 2:00 to 3:00 a.m. and waking up around 11:00 a.m. to 1:00 p.m.
(B) The lower part is about a 5-week period during treatment with APZ 3 mg/day, blue light, and sleep hygiene guidance. From the next morning after starting the treatment, the patient got up around 6:00 to 7:00 a.m. and went to bed around 9:00 to 10:00 p.m., and his sleep rhythm became faster than before the treatment.

APZ, aripiprazole; DSPS, delayed sleep phase syndrome.

**Figure 3:**
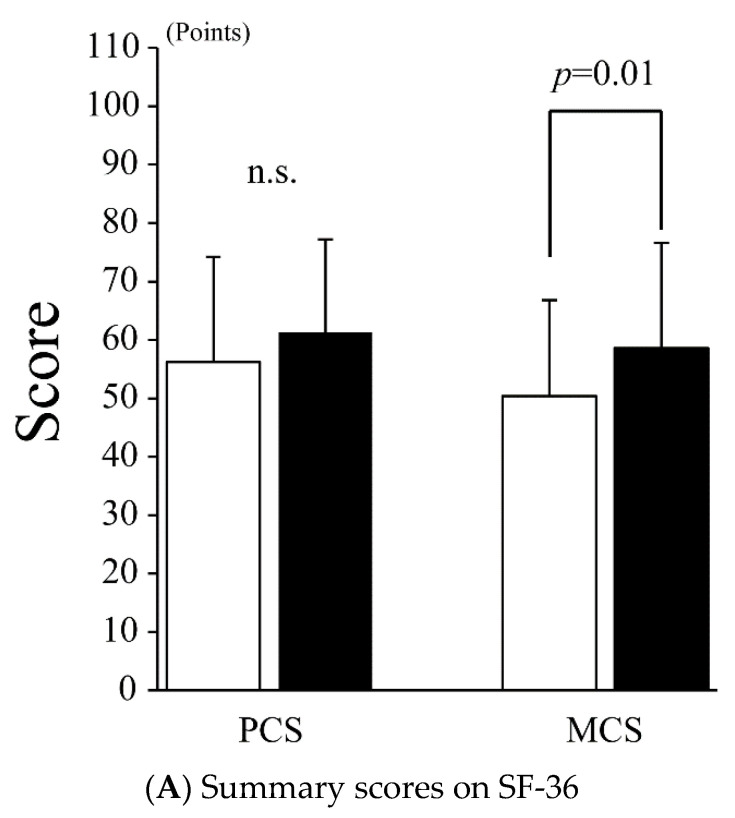

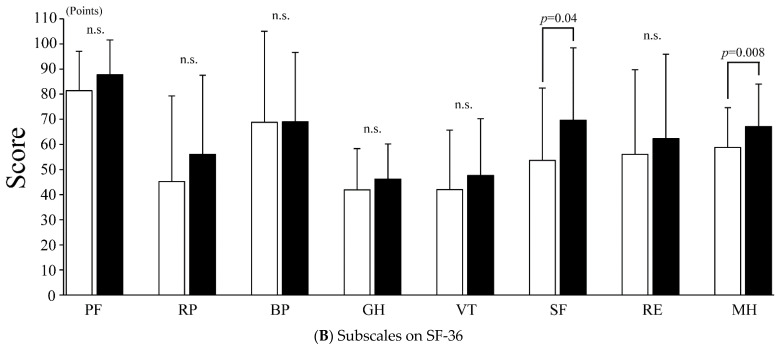
Quality of life by SF-36. (**A**) SF-36: 36-item short-form health survey; PCS: physical component summary; MCS: mental component summary. The white and black bars indicate the values before and after treatment, respectively. Values are presented as the mean ± SD. Error bar represents SD. (**B**) SF-36: 36-item short-form health survey; PF: physical functioning; RP: role-physical; BP: bodily pain; GH: general health; VT: vitality; SF: social functioning; RE: role-emotional; MH: mental health. White and black bars indicate values before and after treatment, respectively. Values are presented as the mean ± SD. Error bar represents SD. n.s., not significant.

Regarding QOL according to the SF-36, as shown in Figure 3A, the PCS did not improve after treatment (56.2 ± 18.0 vs. 61.3 ± 15.9 points, *p* = 0.13), while the MCS improved significantly after treatment (50.4 ± 16.4 vs. 58.6 ± 18.0 points, *p* = 0.01). In addition to the SF-36 summary score, significant improvements were observed in SF and MH (SF: 53.6 ± 28.8 vs. 69.6 ± 28.9 points, *p* = 0.04; MH: 58.8 ± 15.8 vs. 67.1 ± 16.9 points, *p* = 0.008) (Figure 3B), while other SF-36 subscales, such as PF, RP, BP, GH, VT, and RE, did not improve after treatment (PF: 81.4 ± 15.7 vs. 87.7 ± 13.9, *p* = 0.06; RP: 45.2 ± 34.1 vs. 56.0 ± 31.5, *p* = 0.13; BP: 68.8 ± 36.2 vs. 69.0 ± 27.6, *p* = 0.71; GH: 41.9 ± 16.4 vs. 46.2 ± 14.0 points, *p* = 0.22; VT: 42.0 ± 23.7 vs. 47.6 ± 22.6 points, *p* = 0.18; and RE: 56.0 ± 33.7 vs. 62.3 ± 33.6 points, *p* = 0.33).

## 4. Discussion

To the best of our knowledge, this retrospective study is the first to examine sleep hygiene guidance, low-dose APZ administration, and BL exposure combination therapy in teenage patients with wake-up difficulty.

In recent years, the number of non-school-attending teenagers with wake-up difficulties has rapidly increased, as previously mentioned. Our previous case report revealed that low-dose APZ and BL combination therapy was effective in a patient with wake-up difficulty due to CRSWD and prolonged sleep time [11]. Based on this report on APZ administration and BL exposure in patients with wake-up difficulty, this study added sleep hygiene guidance, thus, developing a novel combination therapy. In teenage patients with wake-up difficulty, our combination therapy improved wake-up difficulty by a rate as high as 85.7% and further improved school non-attendance by 66.7%. In addition, significant improvements in the depressive state (SDS) and mental QOL (SF-36) were also observed, suggesting that the combination therapy is substantially effective for wake-up difficulty (Table 3 and Figure 2).

The causes of wake-up difficulty commonly include chronic sleep insufficiency, delayed sleep phase, and OD. Although the AASM-recommended sleep time is 8–10 h for people aged 13–18 years [17], the average sleep time in Japan is as short as 7 h 14 min for people aged 13–14 years [18]. Regarding the delayed sleep phase, although circadian rhythms naturally recede during adolescence, many young Asians experience delayed bedtime, and the total sleep time in young Asians is less than that of young people in North America and Europe [19]. Therefore, many young Asians, including Japanese, are expected to suffer from chronic sleep insufficiency associated with a delayed sleep phase. OD is characterized by a dysfunctional autonomic nervous system and presents with various symptoms, such as dizziness and headache, as previously mentioned [2]. It is diagnosed by the Japanese clinical guidelines for juvenile OD [2]. These OD symptoms have commonly been observed in adolescence, and 30–40% of non-school-attending students had OD [2]. DSPS is characterized by a sleep–wake rhythm that recedes for >2 h relative to the normal rhythm. The prevalence of DSPS in adolescents and young adults is 7–16% [20,21]. The prevalence of OD in patients with circadian rhythm sleep disorder is 57.9%, and it is as high as 70% in patients below 20 years of age [22], suggesting that the coexistence of a delayed sleep phase and OD should be considered. Additionally, a delayed sleep phase is often accompanied by mood disorders and depressive symptoms, especially in adolescence [23]. In this study, 20 patients (95.2%) had an SDS ≥ 40, suggesting mild depressive states and social anxiety.

In this study, the average subjective total sleep time in the 21 patients was 10.3 h, and 85.7% of the students required >10 h of sleep. However, the students and their parents were unaware that 6–8 h of sleep would result in sleep insufficiency, as the students required >10 h of sleep. Individualized sleep hygiene guidance, including taking medications at scheduled times and ensuring adequate sleep at appropriate times, was necessary because parents and teachers were not informed about the delayed sleep phase and the associated relative sleep insufficiency. Furthermore, it is necessary to examine not only OD but also sleep disorders in wake-up difficulty because OD, delayed circadian rhythm, and narcolepsy predominantly feature in adolescence. The above awareness changes and sleep studies are key to improving sleep hygiene and school non-attendance due to wake-up difficulty.

APZ is a second-generation antipsychotic that acts as a partial agonist of dopamine D2 and/or D3 receptors, which affect the circadian rhythm [24,25]. The pharmacological action of APZ depends on the sensitivity of the D2/3 receptors, affected by the dose of APZ. APZ, which is currently approved and sold in more than 60 countries, is effective against schizophrenia and irritability associated with autism spectrum disorders, improves the manic symptoms of bipolar disorder, and can serve as an augmentation treatment for major depressive disorder [26]. APZ is a partial agonist of dopamine and serotonin, which at low doses, acts as an antagonist of the dopamine D2 receptor and also regulates serotonin via the 5-HT-1A receptor [27,28]. Additionally, low-dose APZ acts as a D3 agonist and regulates environmentally adapted behavioral motivation [29]. It also inhibits glycogen synthase kinase 3 (GSK-3) [30]. It is considered that such pharmacological action of APZ causes a phase advance effect, shortening of the ciracadian rhythm, shortening of the nocturnal sleep time, and promotion of sleep and wakefulness [31]. Subsequent studies since 2010 have reported the effect of low-dose APZ (0.5–3 mg) in cases in which the sleep phase receded [10,32,33,34,35]. Therefore, it has been suggested that APZ is effective for circadian rhythm sleep–wake disorders, depression, and wake-up difficulty. On this premise, as well as based on our clinical experience [11], APZ was primarily administered at a dose of 3 mg and was expected to be useful for delayed sleep phase and long sleepers with wake-up difficulty and mild depressive states. Furthermore, it was expected that APZ would be able to awaken patients so that they could adapt to social life by shortening sleep time and advancing the circadian rhythm.

Bright morning light is useful in synchronizing social time and the circadian rhythm by shifting the phase in which the circadian rhythm recedes [36]. Furthermore, it has been clarified that its effects on melatonin and brain waves differ, depending on the light wavelength, and that short-wavelength light, such as BL, affects the circadian rhythm [37,38,39]. Based on these findings, BL therapy was considered an effective means of accelerating a regressed circadian rhythm.

Sleep hygiene guidance, low-dose APZ, and BL are effective treatments for a delayed sleep phase, as described above. Therefore, based on our experience regarding the combination therapy of APZ and BL and its associated favorable results, we developed a combination therapy of these three treatments. In this study, wake-up difficulty in the teenage students was considered to be caused not only by OD but also by the addition of a delayed sleep phase to their long sleep times, with an average of 10.3 h. Therefore, we speculated that combination therapy involving three approaches, namely, cognitive-behavioral therapy with sleep hygiene guidance, drug therapy with low-dose APZ, and time-biological therapy with BL, would be effective in teenage patients with wake-up difficulties. The combination therapy improved wake-up difficulty at a high rate because, in addition to appropriate sleep hygiene guidance, low-dose APZ shortened nocturnal sleep time, promoted wakefulness timing, and induced awakening by suppressing melatonin secretion by BL; moreover, the circadian rhythm was considered to have adjusted to promote awakening at an appropriate time in the morning. Because the patients experienced mild depressive states before treatment, it was important to improve not only the rhythm and length of nocturnal sleep but also the mental QOL of teenagers during the period of physical and mental growth. The results suggest that the combination therapy potentially improves mental health and social life by synchronizing the circadian rhythm with the external rhythm. To evaluate the therapeutic effect on patients with wake-up difficulty, it is apparently necessary to evaluate not only the sleep–wake rhythm but also sleep quality and mental QOL before and after treatment.

This study had some limitations. In this study, we cannot deny selection bias exists because of the nature of retrospective study. The number of cases in this study was small, the data were from a single institution, and only Japanese patients were targeted. Hence, an accumulation of cases in the future is warranted. We evaluated the therapeutic effects 4 weeks after the treatment. Therefore, another limitation of this study was that the therapeutic effects more than 1 month after the treatment were unknown. We would like to carefully observe the progress of the participants to evaluate the long-term treatment effects. It is also necessary that the appropriate APZ dosage and dosing time are considered. We intend to examine the potential factors underlying the cases that do not favorably respond to combination therapy and implement measures to improve the therapy so that patients experiencing wake-up difficulty can be more effectively treated in the future. Additionally, to accurately evaluate the therapeutic effect, comparisons with a control group, placebo group, or groups of each diagnosis are necessary, but we did not include these groups in this study because the number of cases was small. Moreover, some patients had multiple disorders, such as idiopathic hypersomnia and delayed sleep–wake phase disorders. Because of the patients’ nature, we could not purely assess the effects of our intervention in some of them. We would like to investigate this in the future. In this study, we calculated the subjective sleep time from each patient’s sleep-diary records, but in future studies, more objective assessment using actigraphy would be preferred.

## 5. Conclusions

This study indicated that wake-up difficulty was caused by the addition of a delayed sleep phase to students’ long sleep times, with an average of 10.3 h. Furthermore, the novel combination therapy involving sleep hygiene guidance, low-dose APZ administration, and BL exposure was effective in reducing nocturnal sleep time and stabilizing circadian rhythms, thus, improving wake-up difficulty and mental QOL.

## Figures and Tables

**Table 1:** Clinical characteristics before the combination therapy.

Clinical Characteristics	
Number	21
Age, years	14.8 ± 1.7
Male, N (%)	9 (42.9)
School, N (%)	
Junior high school	15 (71.4)
High school	6 (28.6)
Underlying diseases, N (%)	
ADHD	3 (14.3)
Hypopituitarism	1 (4.5)
Medications, N (%)	
Midodrine hydrochloride	3 (14.3)
Atomoxetine	1 (4.8)
Thyradin	1 (4.8)
Questionnaires at the first visit	
ESS ≥ 11 points, N (%)	6 (28.6)
PSQI ≥ 5.5 points, N (%)	12 (57.1)
SDS ≥ 40 points, N (%)	20 (95.2)
Sleep diary	
Subjective sleeping time, hour	10.3 ± 1.0
9-h sleep, N (%)	3 (14.3)
10 h sleep, N (%)	13 (61.9)
11-h sleep, N (%)	1 (4.8)
12-h sleep, N (%)	4 (19.0)

Abbreviations: N, Number; BMI, body mass index; ADHD, attention-deficit hyperactivity disorder; ESS, Epworth Sleepiness Scale; PSQI, Pittsburgh Sleep Quality Index; SDS, Self-Rating Depression Scale; AQ, autism-spectrum quotient.

**Table 2:** Sleep structures after the combination therapy.

Sleep Studies	
PSG	
Stage N1, %	20.1 ± 15.0
Stage N2, %	45.8 ± 10.4
Stage N3, %	16.7 ± 6.2
REM sleep, %	17.5 ± 6.3
Total sleeping time, min	501.8 ± 52.9
Sleep efficiency, %	87.4 ± 8.4
AHI ≥ 5/h, N (%)	2 (9.5)
MSLT	
MSL, min	10.1 ± 4.2
MSL ≤ 8 min, N (%)	6 (28.6)
SOREMP, N (%)	2 (9.5)

Abbreviations: PSG, polysomnography; REM, rapid eye movement; AHI, apnea-hypopnea index; PLMI, periodic limb movement index; MSL, mean sleep latency; SOREMP, sleep-onset rapid eye movement sleep.

**Table 3:** Effects of the combination therapy on subjective sleep quality and depression.

	Before	After	*p*-Value
Patients with wake-up difficulty, N (%)	21 (100)	3 (14.3)	
Non-school-attending patients, N (%)	21 (100)	7 (33.3)	
Subjective total sleeping time, hours	10.3 ± 1.0	9.5 ± 0.6	0.0004
Subjective sleep qualities, points			
ESS	7.7 ± 4.5	7.3 ± 5.4	0.77
Insomnia, depression, points			
PSQI	6.7 ± 2.8	5.3 ± 2.1	0.07
SDS	48.0 ± 6.6	43.5 ± 7.2	0.002

Abbreviations: ESS, Epworth Sleepiness Scale; PQSI, Pittsburgh Sleep Quality Index; SDS, Self-Rating Depression Scale.

## Data Availability

Data sharing does not apply.
